# Dry Period or Early Lactation—Time of Onset and Associated Risk Factors for Intramammary Infections in Dairy Cows

**DOI:** 10.3390/pathogens10020224

**Published:** 2021-02-18

**Authors:** Julia Nitz, Nicole Wente, Yanchao Zhang, Doris Klocke, Martin tho Seeth, Volker Krömker

**Affiliations:** 1 Department of Microbiology, Faculty of Mechanical and Bioprocess Engineering, University of Applied Sciences and Arts, 30453 Hannover, Germany; Julia-Nitz@web.de (J.N.); nicole.wente@hs-hannover.de (N.W.); yanchao.zhang@hs-hannover.de (Y.Z.); doris.klocke@hs-hannover.de (D.K.); martin.tho-seeth@hs-hannover.de (M.t.S.); 2 Department of Veterinary and Animal Sciences, University of Copenhagen, 1870 Frederiksberg C, Denmark

**Keywords:** dairy cows, intramammary infection, mastitis, risk factor, early lactation, dry period

## Abstract

The aim of this study was to define the time-related period of intramammary infections and its relation to risk factors for intramammary infections and clinical mastitis at cow and quarter levels. In total, 269 German Holstein Frisian dairy cows on three farms in Northern and Eastern Germany were included in this study. Quarter milk samples were collected at dry-off, 3 ± 1 days after calving and 17 ± 3 days after calving, for cytomicrobiological examination. Risk factors at quarter- and cow-level associated with intramammary infections and clinical mastitis were recorded during the trial period. Data were analyzed using logistic regression procedures and odds ratios were calculated. Calving for the second time increased the odds of clinical mastitis during the first 100 days of lactation compared to cows calving for the third time or more. A high milk yield after calving was a risk factor for new infections, with environmental pathogens 17 ± 3 days postpartum. A body condition score after calving less than 3.5 was associated with a decreased risk of having an intra-mammary infection (IMI) with non-*aureus* staphylococci and coryneforms 3 ± 1 days postpartum and consistent body condition between dry-off and early lactation decreased the risk of intramammary infections after calving. The absence of a ring of hyperkeratosis at the teat apex shown at dry-off was associated with a lower risk of intramammary infections with environmental pathogens 17 ± 3 days postpartum. This study shows the important influence of the dry period and early lactation on intramammary infections and clinical mastitis postpartum in dairy cows. Udder quarters may have eliminated pathogens during the dry period in 43.6% of cases in this study. Additionally, new infections occurred during early lactation, so 5.1% more quarters were infected 17 ± 3 days compared to 3 ± 1 days postpartum. New infections can be traced to non-*aureus* staphylococci and *Staphylococcus aureus* from dry-off up until 3 ± 1 days postpartum, and to non-*aureus* staphylococci, *Staphylococcus aureus* and *Streptococcus uberis,* after calving. In total, 88.7% of the infected quarters showed new infections with another pathogen species 3 ± 1 days postpartum than at dry-off, and 89.2% of the quarters 17 ± 3 days postpartum than 3 ± 1 days postpartum. In conclusion, the early lactation has just as important an influence on intramammary infections postpartum in dairy cows as the dry period. There is the possibility that udder quarters eliminate pathogens during the early lactation, especially during the dry period. However, there is also the danger that new infections manifest, with a large proportion of new infections occurring after calving. Thus, additional control strategies are of great importance to prevent new infections occurring during early lactation as well as during the dry period to reduce negative effects on milk yield and culling hazards in dairy cows by minimizing the associated risk factors.

## 1. Introduction

As one of the most important and costly diseases on dairy farms, mastitis causes both economic loss and milk quality reduction [1,2,3]. Inherently, mastitis may occur at any stage of the reproductive cycle. Nevertheless, it is well established that, at dry-off and around calving, the mammary glands are more susceptible to the invasion of udder pathogens [4]. Therefore, there are moments of an increased relationship between new intramammary infections (IMI) and resulting mastitis.

The importance of the dry period has been well described [5,6,7,8]. Most infections in the dry period are caused by environmental bacteria, like *Streptococcus uberis* and *Escherichia coli*. [9]. IMI existing during the dry period may be persistent cases from the lactation or may have occurred between dry-off and calving [10]. The occurrence of IMI during the dry period poses an increased risk of clinical mastitis (CM) after calving in the subsequent lactation [11]. Furthermore, CM associated with IMI in the dry period occurs earlier after calving than CM in cows with no IMI during the dry period [12].

Early lactation immediately after calving is described as another moment of great importance for the infection of the mammary glands [3]. The main amount of the infections is caused by *Corynebacterium* spp., non-*aureus* staphylococci and pathogens found in the cow’s environment, like coliform bacteria [13,14].

To date, detecting the precise moment in the reproductive cycle when the infection of the mammary glands occurs in the field has been inconclusive. Information on the presence of inflammation is provided on a monthly basis by the dairy herd improvement (DHI) record. In studies (e.g., [15,16]) and in practice, the rate of new infections and healing of mastitis during the dry period recorded in the DHI tests is used to characterize the dry cow management of a herd by pointing out the percentage of cows with a somatic cell count (SCC) > 100,000 cells/mL (≤100,000 cells/mL) at the first test after calving of all cows with ≤100,000 cells/mL (>100,000 cells/mL) at dry-off in the herd. This makes herds comparable, but it is not possible to evaluate the moment of infection and the responsible risk factors because the timeframe between the last testing and dry-off differs between animals, as does the timeframe between calving and the first day of testing. Furthermore, only the dry period is evaluated.

Previous research was unable to date the precise moment when the mammary glands were infected, with many papers already showing the importance of infections during the dry period (see above). However, the possibility of infection arising during early lactation has as yet not been verified because milk samples were collected only once after calving and not within a tight timeframe [7,9,12,17]. A time interval of more than 14 days between two cases of CM might be a sign of recurrent CM [18]. Thus, a sampling scheme with two samples, with an interval of 14 days between them, allows for the separation of an IMI in the second sample from IMI in the first sample and entails the risk of misinterpretation of contaminations as IMI.

Nonetheless, restricting the relevant timeframe is of particular importance for the prevention of mastitis, as CM is a multifactorial disease and can be affected positively by detecting and controlling predisposing factors [2]. The incidence of mastitis depends on three components: exposure to microbes, udder defense mechanisms, and environmental risk factors [3].

Several risk factors for IMI and CM have been described to date. These can be divided into those at herd-level (e.g., barn cleaning: [3]; season: [19]); cow-level (e.g., hyperketonemia: [20]; claw health: [21]; body condition score: [22]; udder hygiene: [23]; milk yield at dry-off: [24]) and teat-level (e.g., hyperkeratosis: [25]). The purpose of this research was to consider the associated factors, divided into the timeframe between dry-off and calving, from early lactation.

Therefore, the aim of this study was to provide a more precise timeframe for IMI occurring in dairy cows during dry-off and early lactation, and to align this with risk factors for IMI. This would enable a targeted prevention of new infections of the mammary glands and resulting mastitis by influencing known risk factors during the sensitive phase of the reproductive cycle. Furthermore, the adverse impact of IMI on further udder health and milk production would be hindered.

## 2. Results

### 2.1. Numbers of Cows and Quarters

In total, 269 clinically healthy dairy cows at the day of clinical admission were included in the study on the three farms. Of these cows, 21 were excluded before the study was completed. These animals left the study because of too few submitted samples. Additionally, 32 single quarters of the remaining cows were no longer included in the study owing to parenchymal damage and loss of the quarter at the time of one of the quarter milk samples. Therefore, finally, 960 udder quarters were evaluated. The number and percentage of cows and quarters included in the analyses for assessing the risk factors are given in Table 1. Table 2 gives an overview of the mean, standard deviation, median and range for categories of independent variables, with continuous distribution of the risk factors for intramammary infections and clinical mastitis during the dry period and early lactation.

### 2.2. Bacteriological Examination

In total, 960 quarters were cytomicrobiologically examined at drying off, 3 ± 1 days after calving and 17 ± 3 days after calving. The results are shown in Table 3.

The percentage of quarters with no detected pathogen amounted to 60.2% (*n* = 582) at drying off, 87.1% (*n* = 836) 3 ± 1 days after calving and 82.6% (*n* = 793) 17 ± 3 days after calving, respectively. Thus, 43.6% more quarters were non-infected 3 ± 1 days after calving compared to dry-off. However, 5.1% more quarters were infected 17 ± 3 days after calving compared to 3 ± 1 days after calving. In 39.4% (*n* = 378) of the quarters, IMI was detected at drying off; non-*aureus* staphylococci (NAS) constituted the most frequently isolated pathogen (*n* = 159, 16.6%), and 11.0% (*n* = 106) of cases were infected with *Corynebacterium* spp., and 4.6% (*n* = 44) of cases with more than one pathogen. Three ± 1 days after calving, 12.9% (*n* = 124) of all quarters were infected. NAS (*n* = 48, 5.0%), *Pseudomonas* spp. (*n* = 17, 1.8%) and *Staphylococcus aureus* (*n* = 16, 1.7%) were found in high percentages. Seventeen ± 3 days after calving, IMI was detected in 17.4% (*n* = 167) of the quarters. A total of 8.6% (*n* = 83) of the quarters were infected with NAS, 1.7% (*n* = 16) with *Staphylococcus aureus*, 1.6% (*n* = 15) with *Streptococcus uberis* or *Corynebacterium* spp. or more than one pathogen.

### 2.3. Ratio of New Infections to Persistent Infections of the Samples with Pathogens Detected 3 ± 1 and 17 ± 3 Days Postpartum

Table 4 and Table 5 show the ratio of new infections to persistent infections of the 124 samples with pathogens detected 3 ± 1 days postpartum and of the 167 samples 17 ± 3 days postpartum. Of these 124 quarters with pathogens detected 3 ± 1 days postpartum, 14 (11.3%) showed persistent infections with the same pathogen species, especially NAS, at drying off and 3 ± 1 days postpartum. A total of 110 (88.7%) of the quarters showed new infections with another pathogen species 3 ± 1 days postpartum than at drying off. NAS, *S. aureus* and *Pseudomonas* spp. constituted a major percentage of the pathogen species 3 ± 1 days postpartum. The 110 newly infected quarters 3 ± 1 days postpartum resulted from 58 quarters with no detected pathogen and 52 quarters with detection of another pathogen species at drying off.

A total of 18 (10.8%) of the 167 quarters with pathogens detected 17 ± 3 days postpartum showed persistent infections with the same pathogen 3 ± 1 days postpartum, especially NAS. A total of 149 (89.2%) of the infections occurring after 3 ± 1 days postpartum were caused by NAS, *Staphylococcus aureus* and *Streptococcus uberis*. The 149 newly infected quarters 17 ± 3 days postpartum were derived from 128 quarters with no detected pathogen and 21 quarters with another detected pathogen 3 ± 1 days postpartum.

A total of 63 cases of CM were detected during the first 100 days of lactation. An overview of the culture results is given in Table 6. In 15 of the 63 cases of CM, no pathogen was verifiable. In five cases, the same pathogen was identified (*Streptococcus uberis*) in both samples, and in 43 of the 63 cases, a new infection was demonstrable 17 ± 3 days after calving. A total of 10 of these 43 quarters showed an infection with a different pathogen 17 ± 3 days after calving, and in 33 of these quarters with CM, 17 ± 3 days after calving, no pathogen was detected.

### 2.4. Risk Factors in Cows and Quarters Associated with Intramammary Infections and Clinical Mastitis Postpartum

The results of final logistic regression models for the probability of a quarter developing an IMI or CM are given in Table 7. A BCS after calving of less than 3.5 was associated with a decreased risk of having an IMI with NAS and coryneforms 3 ± 1 days postpartum. Cows showing no ring of hyperkeratosis at the teat apex showed a lower risk of IMI with environmental pathogens 17 ± 3 days postpartum. Animals with high milk yield recorded at the first improvement test after calving had a greater risk of IMI with environmental pathogens 17 ± 3 days postpartum. Cows calving for the second time were more likely to have CM during the first 100 days of lactation than cows calving for the third time or more.

## 3. Discussion

This study focused on determining the period of IMI in dairy cows between dry-off and early lactation, and aimed to relate this to risk factors at cow- and quarter-level for IMI and CM. Identifying the moment of infection and risk factors associated with the infection of the udder is necessary to reduce the negative effects of mastitis in cows on milk yield [18] and culling hazards [26].

Infections of a mammary gland can be influenced by external factors because they depend on exposure to microorganisms, udder defense mechanisms and environmental risk factors [3].

IMI manifesting during the dry period can be divided into existing infections (taken over into the dry period from the previous lactation) and new infections (occurring between the time of drying off and calving) [6]. Green et al. [12] stated that mammary glands infected during the dry period are at greater risk of developing CM during subsequent lactation compared to uninfected glands. Moreover, they assumed that CM in cows with an IMI in the previous dry period with the same pathogen occurred more promptly postpartum than CM in cows without IMI with the same pathogen during the dry period. In their study, an IMI in the late dry period increased the risk of CM with the same pathogen; 38.1% of the CM were affected by a pathogen already detected during the dry period. However, it was not possible to specify whether this was a new infection or an infection which had persisted in the udder quarter since the dry period; they suspected a persistence of pathogens from the dry period to CM. The present study showed that approximately 44% quarters were less infected 3 ± 1 days after calving compared to the dry-off. Nevertheless, new infections happened during this timeframe, with these amounting to 89% in relation to persistent infections of about 11%. Nearly the same ratio of new infections and persistent infections was detected in the timeframe of 3 ± 1 days and 17 ± 3 days postpartum. The sampling scheme permits a clear allocation of new and persistent infections; only a distinction between persistent infection and reinfection is impossible. Wente et al. [27] differentiated pathogen species in cases of recurrent CM by means of Random Amplified Polymorphism DNA-Polymerase Chain Reaction. They confirmed a contribution of new infections as compared to persistent infections in cases of recurrent CM. Thus, it is conceivable that a part of the supposed 11% persistent infections were reinfections with the same pathogen as before, and that ratio changed even more to the benefit of new infections. Additionally, most cases of persistent infections showed the isolation of NAS and *Corynebacterium* spp. These pathogens were evaluated as a group of species or at the genus level, respectively. Thus, the proportion of new infections might be even higher because these pathogens were not identified at the species or strain level. Reasons for renewed infections of the same quarter may be the susceptibility of certain quarters to IMI, an increase in the risk of subsequent infections because of previous infection or damage to the mammary environment and reduced innate defense mechanisms because of previous infection [12].

Due to the contribution of new infections as compared to persistent infections between the dry off period and 17 ± 3 days after calving, more focused prevention of new infections should occur during the dry period, especially for early lactation.

For the present study, cows from three Northern German dairy farms were examined. The farms were randomly selected regarding their willingness to participate in the study. Therefore, there was no representative selection. With regard to the number of cows included in the study, this selection criterion seems sufficient to transfer the results to cows from other farms.

The first sample was determined at the day of dry-off; therefore, the status of infection before this significant period was recorded. The date of the second sampling was chosen as close as possible to the calving day to map the status of infection at the time of this event. Sampling closer to calving than 3 ± 1 days postpartum would influence the results of examination because of the characteristics of the colostrum [28]. Colostrum is characterized by a yellowish color and a viscous consistency. The duration of the period of colostrum differs between cows. The exact time of sampling was chosen by observation, when the properties of colostrum were no longer maintained. However, the timeframe was limited to 3 ± 1 days postpartum. The third sampling was set, with a 14-day interval from the first sampling. Therefore, the present IMI in the third sample can be separated from IMI in the second sample. Gröhn et al. [18] describe a time interval greater than 14 days between two cases of CM as an indication for a new case.

The IMI-status of cows was determined at dry-off and in early lactation by bacteriological examinations, and so the time of infection was narrowed down. To date, the results of the last DHI -record prepartum and the first postpartum have formed the basis for the inflammation status. The date of the first DHI record after calving depends on the day of calving and the chosen DHI sampling scheme. Hereby, a distinction between infections up until the period of colostrum and the time afterwards is not possible. Our results showed that 89.2% of IMI 17 ± 3 days postpartum were non-existent 3 ± 1 days postpartum, and therefore must be considered new infections. Approximately 88.7% of IMI 3 ± 1 days postpartum were new infections, occurring since the dry-off period.

### Prevalence and Risk Factors for IMI

There seems to be an association between the difference in body condition between dry off and 17 ± 3 days after calving and IMI postpartum. Cows without a loss of body condition during this timeframe are at lower risk of IMI compared to cows with a loss of body condition. A loss of body condition is connected with the development of ketosis [29], and Hillreiner et al. [20] assert an increased β-hydroxybutyrate concentration as partially responsible for an increased mastitis susceptibility during early lactation. An explanation for this is the impairment of the innate immune function in the mammary gland.

Additionally, it could be shown that a BCS less than 3.5 after calving is negatively associated with IMI with NAS and coryneforms between day 3 ± 1 postpartum. Valde et al. [30] assumed that cows with a lower incidence of mastitis infections had significantly lower BCS in the final month before calving and the first month of lactation than those animals in herds with higher infection rates. An explanation for this might be a larger loss of body condition of cows in a fatter condition during the early lactation. Additionally, those cows with a lower BCS after calving showed rarer udder edema than cows with a BCS greater than 3.5 after calving. Nitz et al. [31] demonstrated the same occurrence in heifers, with prolonged udder edema related to an increased risk of developing IMI with NAS and coryneforms three days after calving. Piepers et al. [32] also identified udder edema as an important risk factor for IMI, with contagious major pathogens in heifers. Waage et al. [33] give a possible explanation for the relationship between udder edema and udder health: a defect in the local blood circulation which impairs the immune response in the udder tissue, and the inflammation disturbs the circulation in the udder tissue and causes edema. Krömker et al. [34] identified a greater risk of open teat canals ante partum in heifers with udder edema, with open teat canals increasing the risk of infections [35]. In the present study, an association between udder edema and the occurrence of mastitis was not found.

Furthermore, a higher milk yield in cows and heifers after calving is associated with an infection with NAS [36,37]. Piepers et al. [36] explained this as a protective effect of the IMI with NAS against IMI with other pathogens. They also assumed that cows with IMI with NAS have a decreased incidence of CM and, subsequently, less loss of milk yield compared to animals without NAS infections. Apart from that, Gröhn et al. [18] assumed that NAS may be detected more often in higher milk-producing cows compared to those animals with a lower milk yield. Following these previous studies, there seems to be an association between the milk yield at the first DHI test after calving and an infection with NAS and coryneforms postpartum. Thus, either an increased milk yield might predispose cows to IMI, or an infection by NAS and coryneforms may be protective against major pathogens that would eventually cause milk losses. In the present study, cows with a lower BCS after calving showed less edema and lower milk yield postpartum, so fewer cases of IMI with NAS and coryneforms were expected.

In contrast to this, the present study shows an association between an increased milk yield at the first DHI test and the occurrence of IMI with environmental pathogens 17 ± 3 days after calving. This corresponds to Suriyasathaporn et al. [38], who stated that cows with higher milk yields are at greater risk of developing CM. An explanation for this risk might be an impairment of immune functions, which is proposed by several authors. For example, Stevens et al. [39] describe several reduced immune functions developed by neutrophils during early lactation, especially the delayed recruitment of neutrophils into the infected udder. This may be of great importance for the severity of IMI with *E. coli* postpartum. Loiselle et al. [40] stated that a lower milk production in early lactation influenced metabolite concentrations in a positive way.

There seems to be an association between the lactation number and the risk of CM during the first 100 days of lactation. Cows in their second lactation are at greater risk of developing CM than animals at a higher lactation number. This is in contrast with previous studies. Cows in the third or larger lactation were 15.4 times more likely to experience recurrent cases of mastitis compared with cows in their first lactation [41]. Green et al. [12] state that quarters from cows of fourth or higher parity were at increased risk of developing CM compared to cows during their third parity or less. De Vries and Marcondes [42] state an average productive lifespan of approximately three to four years in countries with high-producing dairy cows. Here, udder health is one of the relevant reasons for culling. Gröhn et al. [43] reported that mastitis is the disease that most influences culling. Neerhof et al. [44] state that cows with mastitis had 1.69 times greater risk of being culled than a herdmate without mastitis. Furthermore, Bar et al. [26] assert that the risk of a cow being culled within a period of at least two months after any CM case is significantly increased by CM. It might be possible that cows with poor udder health from the outset are culled early and do not reach the third lactation. Further research is needed to ensure this presumed relation.

The period of lactation might play an important role. Cows in early and mid-lactation periods are at higher risk of developing CM than cows in late lactation [38]. Moosavi et al. [2] figured out the differences in the likelihood of CM occurrence for each lactation period between cows in the first, second and later parities, and in different seasons. Therefore, during the early lactation period, heifers developed CM earlier than cows in higher parities did. They concluded that the most important stage of lactation regarding the risk of CM occurrence depends on the parity of cows. Additional influences on the occurrence of CM regarding the lactation stage should be considered to analyze the higher rate of CM during the first 100 days in cows in their second lactation. Perhaps older cows have poorer udder health than younger ones and CM occurs later than 100 d postpartum. In the present study, the stage of lactation was not recorded at the occurrence of CM, so further research is needed to verify this assumption.

It could be shown that the absence of hyperkeratosis at dry-off was associated with lower new infections, with environmental pathogens between 3 ± 1 and 17 ± 3 d postpartum. Former studies describe the association between hyperkeratosis and CM, e.g., [25,45,46]. One plausible explanation for the risk of new infections occurring during the dry period may be the insufficient closure of the teat canal and the presence of environmental pathogens. De Pinho Manzi et al. [47] pointed out that animals with very rough teat end rings have a greater predisposition to IMI. Usually, bacteria enter the udder through the teat end orifice, so the first line of defense against CM is the teat canal. Thus, changes or damage in teat tissue close to the teat canal may reduce its effectiveness in preventing IMI, and support the invasion of bacteria into the udder [48]. A callosity at the teat end may be a factor reducing the closure of the teat canal, thus enabling the invasion of bacteria. Neijenhuis et al. [46], Breen et al. [25] and Paduch et al. [49] describe an association between the presence of environmental pathogens and hyperkeratosis. Therefore, an explanation for IMI after calving with environmental pathogens may be the insufficient closure of the teat canal because of hyperkeratosis at dry off and, as a consequence, IMI with environmental pathogens during the dry period. Thus, hyperkeratosis should be avoided. Mein et al. [50] describe seasonal weather conditions, teat end shape, production level and lactation stage, as well as interactions between milking management and machine factors affecting teat-end hyperkeratosis.

In addition, the roughness of the teats’ orifice poses a risk of obstinate contamination with dirt. Schreiner and Ruegg [51] describe the relationship between udder hygiene and isolated pathogens from milk samples. In their study, poor udder hygiene was associated with increased risk of IMI and subclinical mastitis. Guarins’ [52] study shows that teat skin bacterial counts were significantly greater from teats of cows with poor udder hygiene compared to animals with cleaner udders. Abebe et al. [23] confirm the relation between the status of cows’ udder hygiene and the occurrence of mastitis. There might be an increase in the detection of mastitis in line with the increasing level of bacterial counts on the teat skin.

An association between a SCC after calving ≤100,000 cells/mL and lower incidence of IMI postpartum compared to cows with a SCC after calving >100,000 could have been proven and was to be expected, because somatic cells are a reflection of the inflammatory response to an IMI [53]. An association between the absence of pathogens 3 ± 1 days postpartum and a lower occurrence of new infections between dry off and 17 days postpartum could also have been proven and was to be expected.

## 4. Materials and Methods

The study design was applied and described in a previous study [31].

All applicable guidelines for the care and use of animals were followed. The study was approved by the animal welfare committee of the university (University of Veterinary Medicine Hannover, Foundation; file reference: TVO-2017-V-125). The date when ethical approval was obtained is 10th January 2018. An application for a license for animal testing was not required by the local government due to the study design. The study meets the International Guiding Principles for Biomedical Research Involving Animals (1985).

### 4.1. Characteristics of Herds and Cows

A total of 269 German Holstein Frisian cows reared on three farms located in Northern and Eastern Germany were included in the study. The animals were selected according to them being dried-off between August 2017 and September 2017. All cows that had been dried-off during the indicated timeframe were included in the study. In total, 51, 60 and 158 cows on farms 1, 2 and 3 were enrolled in the trial. All of the herds participated in the local dairy herd improvement (DHI) association programme (Lower Saxony or Saxony-Anhalt, respectively). The average herd size of the three study farms was 160, 221 and 784 lactating cows, the average number of lactations (median) was 3.0 (2.5), 3.1 (2.0) and 3.1 (3.0) for the enrolled cows, and the average bulk milk SCC amounted to 150,000, 180,000 and 260,000 cells/mL during the trial period for farms 1, 2 and 3, respectively. Cows were milked two or three times per day in a herringbone or rotary parlour. All farms utilized blanked dry-cow treatment and internal teat sealants and were equipped with a cubicle housing system.

The herds were selected from a database of the Microbiology group of the Faculty II Mechanical Engineering and Bioprocess Engineering, Hannover University of Applied Sciences and Arts, Hannover, Germany, including farms which are regularly monitored for udder health. The herds were selected on the basis of a willingness to comply with the study protocol.

### 4.2. Quarter Milk Sample

The three farms were visited once a week between August 2017 and May 2018 and in this time-period, quarter milk samples and data on farm management practices and hygiene of animals and barns were collected.

Quarter milk samples of cows were collected three times from all four quarters for SCC and microbiological diagnosis, at dry-off, 3 ± 1 days after calving and two weeks later, at day 17 ± 3 after calving between September 2017 and March 2018, to define the prepartum and postpartum IMI status. The exact timepoint of sampling the first sample after calving was when the period of colostrum was over, i.e., when the secretion showed the properties of milk. All samples were aseptically collected by a veterinarian of the research team during the weekly visits or by the farmers after being instructed by the veterinarian in accordance with the guidelines of the German Veterinary Medical Association [54]. Accordingly, before collection, the apex of each teat was cleaned and disinfected with 70% ethanol and the first three streams of milk were discarded. A total of 10 mL of milk was collected aseptically in sterile plastic tubes with boracic acid for preservation [55]. During the sampling process, disposable gloves were used and disinfected with 70% ethanol in between animals.

The cows were observed by the herdsman and the milkers for any signs of CM after parturition. CM was defined as the presence of typical inflammatory characteristics such as reddening, swelling, pain, a hot udder or abnormal milk appearance, like deviations in color or clotted milk and possible systemic signs like fever and depression. Samples of quarters with a CM were collected by the farmers during the first 100 days of lactation. Quarters with parenchymal damage and loss of quarter at the time of the second or third quarter milk sample were no longer considered in the trial and were excluded from statistical analysis because of missing data.

The milk samples collected by the veterinarian were shipped in a cooling box (4–7 °C) to the laboratory (Department of Microbiology, Faculty of Mechanical and Bioprocess Engineering, Hannover University of Applied Sciences and Arts) for further analysis at the day of sampling. Samples taken by the farmers were stored at 4–7 °C for five days maximum on the farms before being transported to the laboratory for further analysis.

### 4.3. Data Collection

Several risk factors associated with CM at cow- and quarter-level were gathered. Data relating to farm management were recorded using a questionnaire in an interview conducted with the farmers at the end of the trial period. The farming facilities were also measured and evaluated within this timeframe by the researchers. The inspection of the cows and barns regarding hygiene was carried out during the weekly farm visits in the period between dry-off and about 17 ± 3 days after the calving date by a veterinarian of the research team. An overview of potential cow- and quarter-level risk factors can be found in Table 8.

The body condition of the cows was determined eight weeks before the expected calving date and approximately 17 ± 3 days after calving. The visual scoring system developed by Edmonson et al. [56] was used for this purpose. This is based on a five-point scale using quarter point increments, ranging from 1 (emaciated) to 5 (obese).

Milking cups becoming detached because of being kicked off was recorded by the milkers during the daily milking routine. This was a subjective evaluation of cows that kick frequently.

The ease of calving was assessed by the farm personnel and recorded in the DHI record. The scale for ease of calving comprised 1 (unassisted calving/no difficulties), 2 (assistance required/low-grade difficulties), 3 (difficult birth) and 4 (Caesarean section).

The cleanliness of the cows’ udders and thighs was determined at drying off and 17 ± 3 days after calving on a 4-point scale by Schreiner and Ruegg [51]: completely free of dirt or having very little dirt (1), slightly dirty (2), mostly covered in dirt (3), completely covered, caked-on dirt (4).

Ketosis and hypocalcemia after calving were ascertained by clinical signs and recorded by the farmers. The farmers differentiated between “clinical signs” when a medical treatment was necessary or “no clinical signs” of the disease.

Occurring lameness was scored by the veterinarian of the research team during a period between drying off and 17 ± 3 days postpartum according to the locomotion scoring system of dairy cattle by Sprecher et al. [57].

Udder edema was assessed approximately 17 ± 3 days after calving by a veterinarian of the research team. Edema was classified as “existing edema” and “no edema”, so a distinction could be made between a long-lasting (existing 17 ± 3 days after calving) and a temporary (no edema 17 ± 3 days after calving) edema. An edema is characterized by a congestion of the skin. Digital pressure leads to pitting, lasting for few minutes.

Potential risk factors at quarter level can be found in Table 8. The hygiene of the teat skin was scored visually before sampling for the absence or presence of dirt or manure. The teat end was cleaned with a swab and a four-point scale was used to describe the amount of manure and litter sticking to the swab: “Clean” (no manure, dirt or dip), “Dip present” (no manure or dirt), “Small amount of dirt and manure present” and “Larger amount of dirt and manure present”. Cook and Reinemann [58] employed the scale after the preparation procedure prior to milking. In our study, it was used before cleaning the teat end and preparing the sampling at drying off and approximately 17 ± 3 days after calving. Thus, an impression of the cows’ hygiene could be gained.

The teat-end condition was recorded at drying off and day 17 ± 3 after calving. The evaluation was based on a four-point scoring system by Mein et al. [50]. This further developed the scoring system by Neijenhuis et al. [59] and is suitable for field evaluations. Teats with no teat-end hyperkeratosis (N, no ring) are distinguished from teat ends showing callosity roughness. Teat-end hyperkeratosis can be smooth or slightly rough (S, a raised ring with no roughness or only mild roughness and no keratin fronds), rough (R, a raised roughened ring with isolated old keratin fronds extending 1–3 mm from the orifice) or very rough (VR, a raised ring with rough old keratin fronds extending >4 mm from the orifice; the rim of the ring is rough and cracked, giving the teat end a “flowered” appearance) ring.

### 4.4. Laboratory Procedures

The laboratory testing was performed at the microbiological laboratory, Faculty of Mechanical and Bioprocess Engineering, Hannover University of Applied Sciences and Arts. The milk samples were cultured and identified in accordance with the guidelines recommended by the German Veterinary Medical Association [54]. Ten microliters of each milk sample were inoculated onto an esculin blood agar plate (5% defibrinated sheep blood, Oxoid Deutschland GmbH, Wesel, Germany). Plates were analyzed after 24 and 48 h of aerobic incubation at 37 °C. The grown colonies were preliminarily differentiated by means of Gram staining, morphology and cell morphology, hemolysis patterns and esculinhydrolysis.

Gram-positive, catalase positive cocci were categorized as NAS. Gram-positive, β-hemolyzing, catalase-producing cocci colonies were additionally tested for the clumping factor (Staph Plus Latex Kit, DiaMondiaL, Vienna, Austria) to verify *Staphylococcus* (*S*.) *aureus* colonies from NAS. Esculin non-hydrolyzing streptococci were assigned to the Lancefield groups by means of the Strep Latex Kit (DiaMondiaL). Esculin hydrolyzing streptococci were cultured on modified Rambach agar medium to check for ß-D-Galactosidase activity [60]; positive colonies were differentiated as *Streptococcus* (*Sc*.) *uberis,* and negative colonies as *Enterococcus* spp.

Gram-positive irregular rods with Y-shaped cell configuration were identified as *Trueperella* (*T*.) *pyogenes*, if they were β-hemolytic and catalase-negative. Gram-positive, non-hemolytic catalase-positive irregular rods were categorized as coryneforms. Catalase-positive Yeasts and Prototheca were determined by microscopy.

Gram-negative rods were differentiated by their ability to catabolize glucose under aerobic and anaerobic atmosphere (glucose supplemented oxidation-fermentation test medium, Merck KGaA, Darmstadt, Germany) and cytochrome C oxidase production (Bactident Oxidase, Merck KGaA). Cytochrome C oxidase-negative colonies fermenting glucose were cultured on Chromocult^®^Coliform Agar (Merck KGaA) to distinguish *Escherichia* (*E*.) *coli* and other coliforms. Non-motile coliforms were additionally referred to as *Klebsiella* spp. Gram-negative, cytochrome C oxidase-positive bacteria, which metabolized glucose oxidatively, were referred to as *Pseudomonas* spp.

Following the guidelines of the German Veterinary Medical Association [53], the status of milk samples was defined. Thus, if at least one colony of the contagious pathogens *S. aureus*, *S. agalactiae*, *S. dysgalactiae* or *T. pyogenes* was ascertained, or more than 10 colonies of one of the other bacterial species, evidence of a correlation with an IMI was given. A sample was considered contaminated if more than two different bacteria species were identified. The SCC of the milk samples was ascertained by flow cytometry, using the SomaScopeTM Smart (Delta Instruments B.V., Drachten, The Netherlands) by the researchers.

### 4.5. Definitions

Differentiating between persistent and new infections was based on the cultural and biochemical identification of identical and non-identical pathogen species in the quarter milk samples at drying off, 3 ± 1 days after calving and 17 ± 3 days after calving. NAS were evaluated as a group of species.

Persistent infections were defined as IMI when the same pathogen species was detectable in the quarter milk samples at drying off and 3 ± 1 days after calving or rather 3 ± 1 and 17 ± 3 days after calving.

New infections were defined as IMI at dry off and 3 ± 1 days after calving, or rather 3 ± 1 days after calving and 17 ± 3 days after calving, with different pathogen species in the same quarter, or IMI detectable 3 ± 1 or rather 17 ± 3 days after calving in a quarter that was free of IMI at dry off or rather 3 ± 1 days after calving.

Contagious pathogens are transmitted during milking from the infected quarter of one cow to another cow through the milker’s hands, liners or milk. Their main reservoir is an infected udder quarter [61]: *Staphylococcus aureus, Trueperella pyogenes*, *Streptococcus dysgalactiae.* Environmental pathogens are transmitted in stable areas and their main reservoir is the cow’s environment [61]: *Streptococcus uberis*, *Escherichia coli*, *Enterococcus* spp., Coliform bacteria, *Pseudomonas* spp., *Bacillus* spp., Prototheca.

### 4.6. Sample Size Calculation

We performed a cross-sectional study, where the unit of investigation was the cow’s udder quarter. With an estimated prevalence of new infections of 15%, a confidence level of 95% and an accepted error of 5%, a sample of 196 udder quarters was required as a minimum [62]. Due to the study’s framework, we were able to significantly exceed the sample size calculated before the study began, thus improving the power of the study.

### 4.7. Statistical Analysis

For analyzing the dataset, the program SPSS 26.0, Chicago IL, USA was used with the udder quarter as the statistical unit. Associations between IMI during the first 17 ± 3 days postpartum or clinical mastitis during the first 100 days of lactation and risk factors (independent variables) were examined with generalized linear mixed models with logit link and binomial response (logistic regression) after pre-screening for variable selection in univariable analysis.

The relation between dependent and independent variables was tested first by means of the Student’s *t*-test/Wilcoxon test/ANOVA for continuous measurements, with the exception of predictors in the same model, which indicated a correlation of r > 0.70 with one another (Spearman/Kendal’s tau to avoid multicollinearity; for this reason, no variables were excluded). Then, independent variables associated with dependent variables at *p* < 0.10 in the Student’s test and X²-test were submitted to binary logistic regressions with IMI with pathogens, IMI with contagious pathogens, IMI with environmental pathogens, IMI with NAS or coryneforms and mastitis. Quarters with a missing value for one potential risk factor were not taken into account.

Using logistic regression procedures, the association between IMI during the first 17 ± 3 days postpartum or mastitis during the first 100 days of lactation and risk factors (independent variables) was examined, and the dependent binary dichotomous variables contained “New infection from dry off to day 3 ± 1 postpartum/no new infection from dry off to day 3 ± 1 postpartum”, “New infection from day 3 ± 1 to day 17 ± 3 postpartum/no new infection from day 3 ± 1 to day 17 ± 3 postpartum”, “IMI with contagious pathogens/no IMI with contagious pathogens”, “IMI with environmental pathogens/no IMI with environmental pathogens”, “IMI with NAS or coryneforms/no IMI with NAS or coryneforms” and “mastitis/no mastitis”. Herd, cow within herd and quarter within cow were considered as random effects.

A backward stepwise procedure was used to select the final multivariate regression model. Potential risk factors were excluded one by one if *p* > 0.05.

Odds ratios (OR) were calculated to describe the direction of the relationship between dependent and independent variables. OR were determined with 95% confidence intervals (CI 95%) and a statistical significance was set at *p* ≤ 0.05.

## 5. Conclusions

The present study showed the important influence of the dry period and early lactation on IMI and CM postpartum in cows. There is the possibility that udder quarters eliminate pathogens during the dry period (this being proven in 43.6% of cases in this study) as well as the occurrence of new infections during early lactation (5.1% more quarters were infected 17 ± 3 days compared to 3 ± 1 days postpartum). However, there is also the danger that new infections manifest themselves from the dry-off up until 3 ± 1 days postpartum, especially with NAS and *Staphylococcus aureus*. New infections after calving are primarily caused by NAS, *Staphylococcus aureus* and *Streptococcus uberis*. The consequences may be CM. Thus, it would appear necessary to prevent new infections occurring during the dry period and early lactation. As related risk factors for new infections, the BCS after calving, the difference in body condition between dry-off and early lactation, lactation number, absence of hyperkeratosis at dry-off and milk yield after calving were determined. A consistent body condition between dry-off and early lactation is preferable, because no difference in body condition decreases the risk of IMI postpartum. A lower BCS after calving decreases the risk of IMI with NAS and *Corynebacterium* spp. after calving; thus, a lower BCS after calving is preferable. The number of lactations is associated with the risk of CM. Having a higher parity than the second one means less risk of developing CM. High milk yield after calving is responsible for an increased risk of IMI with environmental pathogens. To prevent IMI with environmental pathogens 17 ± 3 days postpartum, efforts should be taken to prevent hyperkeratosis before dry-off. Further studies should point out causalities between risk factors and resulting IMI. The importance of the dry period as the origin of IMI is well described, and control strategies to minimize the risk of IMI during the dry period are well established. The present study clarifies that a large proportion of new infections occurs after calving. Therefore, additional control strategies are needed to prevent new infections occurring during early lactation as well as during the dry period to minimize negative effects on milk yield and culling hazards in dairy cows.

## Figures and Tables

**Table 1:** Number and percentage of cows and quarters included in the analyses concerning the risk factors for intramammary infections and clinical mastitis during dry period and early lactation.

Independent Variable	N (%) of Quarters: 960 Quarters
Cow level	
Body condition before calving	
<3.5	203 (21.15)
≥3.5	392 (40.83)
missing	365 (38.02)
Body condition after calving	
<3.5	627 (65.31)
≥3.5	333 (34.69)
Detaching milking cups because of kicking off (yes)	28 (2.92)
Difference in body condition score	
0	127 (13.23)
positive	113 (11.77)
negative	355 (36.98)
missing	365 (38.02)
Duration of dry period	
<60 days	738 (76.88)
≥60 days	222 (23.13)
Duration of previous lactation	
<305 days	250 (26.04)
≥305 days	710 (73.96)
Ease of calving	
1	704 (73.33)
2	217 (22.60)
3	20 (2.08)
4	0
Missing	19 (1.98)
Hygiene score: cow at drying off	
1 and 2	446 (46.46)
3 and 4	100 (10.42)
missing	414 43.13)
Hygiene score: cow after calving	
1 and 2	716 (74.58)
3 and 4	204 (21.25)
missing	40 (4.17)
Hypocalcemia after calving (yes)	43 (4.48)
Individual access to pasture before or after calving (yes)	268 (27.92)
Ketosis after calving	
yes	35 (3.65)
missing	522 (54.38)
Lameness (yes)	98 (10.21)
Milk yield before drying off	
≤20 L	234 (24.38)
>20 L	718 (74.79)
missing	8 (0.83)
Number of lactations	
2nd lactation	442 (46.04)
≥3rd lactation	518 (53.96)
SCC ^1^ before drying off	
≤100,000 cells/mL ^2^	498 (51.88)
>100,000 cells/mL	454 (47.29)
missing	8 (0.83)
SCC ^1^ after calving	
≤100,000 cells/mL ^2^	672 (70)
>100,000 cells/mL	288 30)
Season of calving	
winter	387 (40.31)
fall	573 (59.69)
Udder edema (yes)	174 (18.13)
missing	273 (28.44)
Quarter level	
Hygiene score: teat skin at drying off	
1 and 2	335 (34.9)
3 and 4	229 (23.85)
missing	396 (41.25)
Hygiene score: teat skin after calving	
1 and 2	455 (47.4)
3 and 4	221 (23.02)
missing	284 (29.58)
Teat apex condition at drying off	
no ring	183 (19.06)
rough	404 (42.08)
missing	373 (38.85)
Teat apex condition after calving	
no ring	186 (19.38)
rough	494 (51.46)
missing	280 (29.17)
Teat lesions (yes)	2 (0.21)

^1^ Somatic Cell Count. ^2^ Milliliter.

**Table 2:** Mean, standard deviation, median and range for categories of independent variables with continuous distribution of the risk factors for intramammary infections and clinical mastitis during dry period and early lactation.

Independent Variable	Mean	Standard Deviation	Median	Range
Duration of previous lactation (days)	346.63	60.29	328	267–597
Duration of dry period (days)	55.83	12.66	54	27–152
Milk yield before drying off (liter)	25.14	7.21	24.7	4.1–43.7
Milk yield after calving (liter)	44.21	8.53	44.35	14.4–69.5
SCC ^1^ before drying off (cells/mL ^2^)	231.12	403.31	91	8–3601
SCC ^1^ after calving (cells/mL ^2^)	301.84	1050.00	42.5	6–11,866
Number of lactations	3.03	1.24	3	2–8

^1^ Somatic Cell Count. ^2^ Milliliter.

**Table 3:** Pathogens isolated from quarter milk samples at drying off, 3 ± 1 and 17 ± 3 days after calving.

Pathogen	Number (%) of Isolated Pathogens		
	At Drying Off	3 ± 1 Days After Calving	17 ± 3 Days After Calving
NAS ^1^	159 (16.4)	48 (5.0)	83 (8.6)
*Corynebacterium* spp.	106 (11.0)	10 (1.0)	15 (1.6)
Contagious pathogens			
*Staphylococcus aureus*	42 (4.4)	16 (1.7)	16 (1.7)
*Trueperella pyogenes*	0	5 (0.5)	2 (0.2)
*Streptococcus dysgalactiae*	5 (0.5)	0	6 (0.6)
Environmental pathogens			
*Streptococcus uberis*	8 (0.8)	5 (0.5)	15 (1.6)
*Enterococcus* spp.	8 (0.8)	2 (0.2)	1 (0.1)
Coliform bacteria ^2^	3 (0.3)	7 (0.7)	10 (1.0)
*Pseudomonas* spp.	3 (0.3)	17 (1.8)	3 (0.3)
*Bacillus* spp.	0	6 (0.6)	0
Prototheca	0	0	1 (0.1)
Mixed	44 (4.6)	8 (0.8)	15 (1.6)
**In total**	378 (39.4)	124 (12.9)	167 (17.4)
**No specific growth**	582 (60.6)	836 (87.1)	793 (82.6)

^1^ Non-*aureus* staphylococci. ^2^ Coliform bacteria: *Escherichia coli*, *Enterobacter*, *Klebsiella* spp.

**Table 4:** Cause of infections existing at day 3 ± 1 postpartum ^1^.

	Infected Quarters 3 ± 1 Days after Calving (% ^4^)	Persistent ^2^ Infections (% ^5^)	New ^3^ Infections (% ^5^)
*Staphylococcus aureus*	16 (1.7)	0	16 (100)
*Streptococcus uberis*	5 (0.5)	0	5 (100)
*Pseudomonas* spp.	17 (1.8)	0	17 (100)
NAS ^6^	48 (5)	10 (20.8)	38 (79.2)
*Corynebacterium* spp.	10 (1)	4 (40)	6 (60)
*Bacillus* spp.	6 (0.6)	0	6 (100)
*Trueperella pyogenes*	5 (0.5)	0	5 (100)
Coliform bacteria ^7^	7 (0.7)	0	7 (100)
*Enterococcus* spp.	2 (0.2)	0	2 (100)
More than one pathogen	8 (0.8)	0	8 (100)
**In total**	124 (100)	14 (11.3)	110 (88.7)
**In total**		124 (100)	

^1^ The differentiation based on cultural and biochemical identification of identical and non-identical pathogen species in the quarter milk samples at drying off and 3 ± 1 days after calving. ^2^ Persistent infections are defined as intramammary infections with the same pathogen species detectable in the quarter milk samples at drying off and 3 ± 1 days after calving. ^3^ New infections are defined as intramammary infections at drying off and 3 ± 1 days after calving with different pathogen species in the same quarter or intramammary infections detecTable 3 ± 1 days after calving in a quarter that was free of intramammary infections at drying off. ^4^ Percentage of one pathogen group of all infected quarters 3 ± 1 days postpartum. ^5^ Percentage of persistent infections or new infections refers to all the infected quarters 3 ± 1 days postpartum. ^6^ Non-*aureus* staphylococci. ^7^ Coliform bacteria: *Escherichia coli*, *Enterobacter*, *Klebsiella* spp.

**Table 5:** Cause of infections existing at day 17 ± 3 postpartum ^1^.

	Infected Quarters 17 ± 3 Days after Calving (% ^4^)	Persistent ^2^ Infections (% ^5^)	New ^3^ Infections (% ^5^)
*Staphylococcus aureus*	16 (1.7)	1 (6.3)	15 (93.8)
*Streptococcus uberis*	15 (1.6)	1 (6.7)	14 (93.3)
*Pseudomonas* spp.	3 (0.3)	0	3 (100)
NAS ^6^	83 (8.6)	11 (13.3)	72 (86.7)
*Corynebacterium* spp.	15 (1.6)	4 (26.7)	11 (73.3)
*Trueperella pyogenes*	2 (0.2)	0	2 (100)
Coliform bacteria ^7^	10 (1)	0	10 (100)
*Streptococcus dysgalactiae*	6 (0.6)	0	6 (100)
*Enterococcus* spp.	1 (0.1)	0	1 (100)
Prototheca	1 (0.1)	0	1 (100)
More than one pathogen	15 (1.6)	1 (6.7)	14 (93.3)
**In total**	167 (100)	18 (10.8)	149 (89.2)
**In total**		167 (100)	

^1^ The differentiation based on cultural and biochemical identification of identical and non-identical pathogen species in the quarter milk samples 3 ± 1 days after calving and 17 ± 3 days after calving. ^2^ Persistent infections are defined as intramammary infections with the same pathogen species detectable in the quarter milk samples 3 ± 1 days after calving and 17 ± 3 days after calving. ^3^ New infections are defined as intramammary infections 3 ± 1 days after calving and 17 ± 3 days after calving with different pathogen species in the same quarter or intramammary infections detectable 17 ± 3 days after calving in a quarter that was free of intramammary infections 3 ± 1 days after calving. ^4^ Percentage of one pathogen group of all infected quarters 17 ± 3 days postpartum. ^5^ Percentage of persistent infections or new infections refers to all the infected quarters 17 ± 3 days postpartum. ^6^ Non-*aureus* staphylococci. ^7^ Coliform bacteria: *Escherichia coli*, *Enterobacter*, *Klebsiella* spp.

**Table 6:** Culture results of milk samples from clinical mastitis postpartum.

Pathogen	Clinical Mastitis Postpartum
*Streptococcus uberis*	23
*Pseudomonas* spp.	2
NAS ^1^	1
*Bacillus* spp.	1
*Trueperella pyogenes*	2
Coliform bacteria ^2^	15
More than one pathogen	4
No specific growth	15
**In total**	63

^1^ Non-*aureus* Staphylococci. ^2^ Coliform bacteria: *Escherichia coli*, *Enterobacter*, *Klebsiella* spp.

**Table 7:** Final logistic regression models for the probability of a quarter developing IMI ^1^ or clinical mastitis.

Dependent Variable	Independent Variable (Reference Category)	β ^3^	SE ^4^	OR ^5^	95% CI ^6^ (OR)	*p*-Value
IMI ^1^ with NAS ^2^ and coryneforms 3 ± 1 days postpartum	BCS ^2^ after calving <3.5 (BCS ≥ 3.5)	−0.793	0.3524	0.453	0.227–0.904	0.025
IMI with environmental pathogens 17 ± 3 days postpartum	“No ring” at teat apex at dry off (roughness at teat apex)	−0.969	0.488	0.380	0.146–0.990	0.048
IMI with environmental pathogens 17 ± 3 days postpartum	Milk yield after calving (covariate)	0.059	0.0274	1.061	1.005–1.120	0.031
Clinical mastitis during the first 100 days of lactation	2nd lactation (≥3rd lactation)	1.214	0.324	3.368	1.783–6.359	0.000
IMI during 3 ± 1 days and 17 ± 3 days postpartum	No difference in BCS ^2^ (negative difference in body condition)	−0.946	0.4418	0.388	0.192–0.527	0.033
IMI during 3 ± 1 days and 17 ± 3 days postpartum	SCC ^7^ after calving ≤100,000 cells/mL ^8^ (>100,000 cells/mL ^8^)	−0.998	0.3070	0.369	0.202–0.673	0.001
New infection between dry off and 17 ± 3 days postpartum	No pathogen detected 3 ± 1 days postpartum (pathogen detected)	−0.704	0.2327	0.495	0.314–0.781	0.002

^1^ Intramammary infection. ^2^ Non-*aureus* staphylococci. ^3^ Regression coefficient. ^4^ Standard error of the mean. ^5^ Odds ratio. ^6^ 95% confidence interval. ^7^ Somatic Cell Count. ^8^ Milliliter.

**Table 8:** Overview of cow- and quarter-level risk factors potentially related to IMI ^1^ in cows.

Risk Factor	Description/Classification	Recording Method	Breakdown Categories
**Cow level**			
Body condition before and after calving	Five-point scale at drying off and 17 ± 3 days after calving	Visual	<3.5 versus ≥3.5
Milking cups becoming detached because of being kicked off	Whether or not the milking cups frequently became detached	Interview *	Yes versus no
Difference in body condition score	Difference at drying off versus after calving	Visual	No difference versus positive versus negative development
Duration of dry period	Duration of dry period	DHI ^2^-records	<60 days versus ≥60 days
Duration of previous lactation	Duration of previous lactation	DHI-records	<305 days versus ≥305 days
Ease of calving	Four-point scale, unassisted-assistance required-difficult birth-Caesarian section	DHI-records *	=4 classes
Hygiene score: cow	Thigh and udder hygiene on four-point scale at drying off and 17 ± 3 days after calving	Visual	Scales 1 and 2 versus scales 3 and 4
Hypocalcemia after calving	Whether or not the cow suffered from hypocalcemia after calving	Interview *	Yes versus no
Individual access to pasture before or after calving	Whether or not cows had access to pasture before or after calving	Interview *	Yes versus no
Ketosis after calving	Whether or not cows suffered from ketosis after calving	Interview *	Yes versus no
Lameness	Whether or not cows were lame during the trial period (scores 2–5)	Visual	Yes versus no
Milk yield before drying off	Last test-day milk yield before drying off	DHI-records	20 L versus >20 L
Milk yield after calving	First test-day milk yield after calving	DHI-records	Continuous
Number of lactations	Number of lactations	DHI-records	2nd versus ≥3rd
SCC ^3^	Last test-day SCC before drying off and first after calving	DHI-records	≤100,000 cells/mL ^4^ versus >100,000 cells/mL
Season of calving	Two seasons of calving: fall (September–November), winter (December–February)	DHI-records	Fall versus winter
Udder edema	Whether or not udder edema existed more than 17 ± 3 days after calving	Visual and palpation	Yes versus no
**Quarter level**			
Hygiene score: teat skin	Teat apex on four-point scale at drying off and 17 ± 3 days after calving	Visual	Scales 1 and 2 versus scales 3 and 4
Teat apex condition	No ring-smooth or slightly rough-rough-very rough at drying off and 17 ± 3 days after calving	Visual and palpation	No ring versus rough
Teat lesions	Whether or not the teat was damaged during the first 17 ± 3 days of lactation	Interview and visual	Yes versus no

^1^ Intramammary infections. ^2^ Dairy herd improvement. ^3^ Somatic Cell Count. ^4^ Milliliter. * Data recorded by the farmers.
